# T cell landscape and dynamics in immunoglobulin light chain amyloidosis before and after daratumumab‐based therapy

**DOI:** 10.1002/ctm2.582

**Published:** 2021-11-29

**Authors:** Yujia Wang, Lushuang Xu, Weijia Zhao, Xiaojie Chen, Lei Wen, Wenbing Duan, Xiao‐Juan Yu, Fu‐ De Zhou, Yang Liu, Jie Hao, Xiaojun Huang, Jin Lu, Qing Ge

**Affiliations:** ^1^ Department of Immunology School of Basic Medical Sciences Peking University. NHC Key Laboratory of Medical Immunology (Peking University) Beijing China; ^2^ BasenByte Biotechnology Co., Ltd. Beijing China; ^3^ Beijing Key Laboratory of Hematopoietic Stem Cell Transplantation Peking University People's Hospital & Institute of Hematology Beijing China; ^4^ Renal Division Department of Medicine Institute of Nephrology Peking University First Hospital, & Renal Pathology Center Peking University Beijing China; ^5^ Renal Pathology Center Institute of Nephrology Peking University Beijing China; ^6^ Key Laboratory of Renal Disease Ministry of Health of China Beijing China; ^7^ Key Laboratory of CKD Prevention and Treatment Ministry of Education of China Beijing China; ^8^ Collaborative Innovation Center of Haematology Soochow University Suzhou Jiangsu China; ^9^ Department of Integration of Chinese and Western Medicine School of Basic Medical Sciences Peking University Beijing China; ^10^ National Key Laboratory of Human Factors Engineering China Astronauts Research and Training Center Beijing China

**Keywords:** bone marrow resident memory T cells, daratumumab, immunoglobulin light chain amyloidosis, single‐cell RNA sequencing, T cells

## Abstract

Amyloid light‐chain (AL) is characterized by the presence of small, poorly proliferating plasma cell clones with the production and deposition of light chains into tissues. T cell changes within the tumour microenvironment in AL are poorly understood. By sequencing at a single‐cell level of CD3^+^ T cells purified from bone marrow (BM) and blood of newly diagnosed AL patients before and after a combination of daratumumab with cyclophosphamide, bortezomib, and dexamethasone (Dara‐BCD), we analysed the transcriptomic features of T cells and found an expansion, activation and type I cytokine upregulation in BM and circulating T cells after the treatment. More prominent changes were shown in CD8^+^ T cells. In particular, we found the presence of CD8^+^ BM resident memory T cells (T_RM_) with high expression of inhibitory molecules in AL patients at diagnosis. After Dara‐BCD, these T_RM_ cells were quickly activated with downregulation of suppressive molecules and upregulation of *IFNG* expression. These data collectively demonstrate that Dara‐based therapy in patients with AL amyloidosis promotes anti‐tumour T cell responses. The similar transcriptomic features of BM and circulating T cells before and after therapy further provide a less invasive approach for molecular monitoring of T cell response in AL amyloidosis.

## Graphical abstract

We performed scRNA‐seq of T cells in patients with AL amyloidosis before and after daratumumab‐based therapy and observed prominent changes in CD8^+^ T cells after treatment. In particular, we found the presence of CD8^+^ bone marrow resident memory T cells (TRM) with high expression of inhibitory molecules in AL patients at diagnosis. After daratumumab‐based treatment, these TRM cells were quickly activated with downregulation of suppressive molecules and upregulation of IFNG expression.

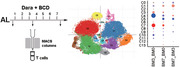

## 1 INTRODUCTION

Amyloid light‐chain (AL) is characterized by the deposition in various organs or tissues of toxic amyloidogenic light chains (LC) produced by abnormal plasma cell (PC) clones in the bone marrow (BM).^1^ The pathogenesis of AL remains less well understood.^2^, ^3^, ^4^ Less than 25% of AL patients with the current mainstay chemotherapy such as a combination of bortezomib, cyclophosphamide and dexamethasone (BCD or CyBorD), and hematopoietic stem cell transplantation achieves a complete and long‐lasting haematological remission and survive for more than 10 years.^5^, ^6^ In particular, AL patients with the most common chromosomal abnormality (*t*(11;14)) had a poor haematologic response and shorter survival when treated with BCD.^7^

Different from other types of plasma cell dyscrasia such as multiple myeloma (MM) and its precursor stage of monoclonal gammopathy of undetermined significance (MGUS) that have well‐characterized numerical and functional alterations in T, natural killer (NK) cells, monocytes/macrophages, and dendritic cells, not much is known of the changes of these cells in AL.^8^, ^9^ Recently, high counts of monocytes and reduced frequencies of NK cells in peripheral blood were found in AL patients and the circulating monocyte counts were associated with an increased risk for AL.^10^, ^11^ The changes in T cells, however, are not clear. The patients with AL had similar frequencies of circulating CD3^+^, CD4^+^ and CD8^+^ cells when compared to healthy controls,^12^ increased ratios of BM CD4^+^ subsets (CCR5^+^ and CD28^+^ subsets) when compared to MM patients.^13^ An increase in the degree of TCR CDR3 skewing was also found in AL patients.^12^ The impact of these seemingly limited T cell alterations on disease progression is not known.^14^ An elevation of CD8^+^ T and a reduction of CD4^+^ T cell frequencies in the BM were observed in MM patients treated with bortezomib, lenalidomide or autologous stem cell transplantation.^9^ Whether these treatments lead to similar changes in patients with AL has not been directly examined.

Daratumumab (Dara) is a CD38‐targeting IgG_1_ antibody that has been approved for MM treatment. In light of the high response rate and improved survival in MM patients with Dara‐containing regimens,^15^, ^16^ it was tested in AL since 2016, initially with relapsed refractory AL and later with newly diagnosed patients.^17^, ^18^ High haematological and organ response rates were reported by various groups, indicating that Dara‐based regimens induce a strong anti‐tumour effect in AL even though MM‐ and AL‐derived PCs showed differences in their transcriptomic features and chromosomal abnormalities.^4^, ^18^, ^19^, ^20^, ^21^, ^22^, ^23^

The treatment with Dara in MM patients has been shown to modulate anti‐tumour T cell response by reducing CD38^+^ regulatory T cells (Tregs) and expanding CD3^+^ T cells (CD8^+^ T cells in particular) with a skewed T cell repertoire.^24^, ^25^, ^26^, ^27^, ^28^ Whether and how Dara‐based therapy affects T cells in AL amyloidosis is not clear. In this study, we characterized the transcriptome of circulating and BM T cells at a single‐cell level in newly diagnosed AL patients before and after Dara‐based therapy (daratumumab with cyclophosphamide, bortezomib and dexamethasone [Dara‐BCD]). We found an expansion, activation and type I cytokine upregulation in BM and circulating T cells with more prominent changes in CD8^+^ T cells after treatment. The presence of CD8^+^ BM resident memory T cells (T_RM_) with unique transcriptome features was further revealed.

## 2 RESULTS

### 2.1 scRNA seq revealed dynamic alterations of T cells in patients before and after Dara‐BCD

**FIGURE 1:**
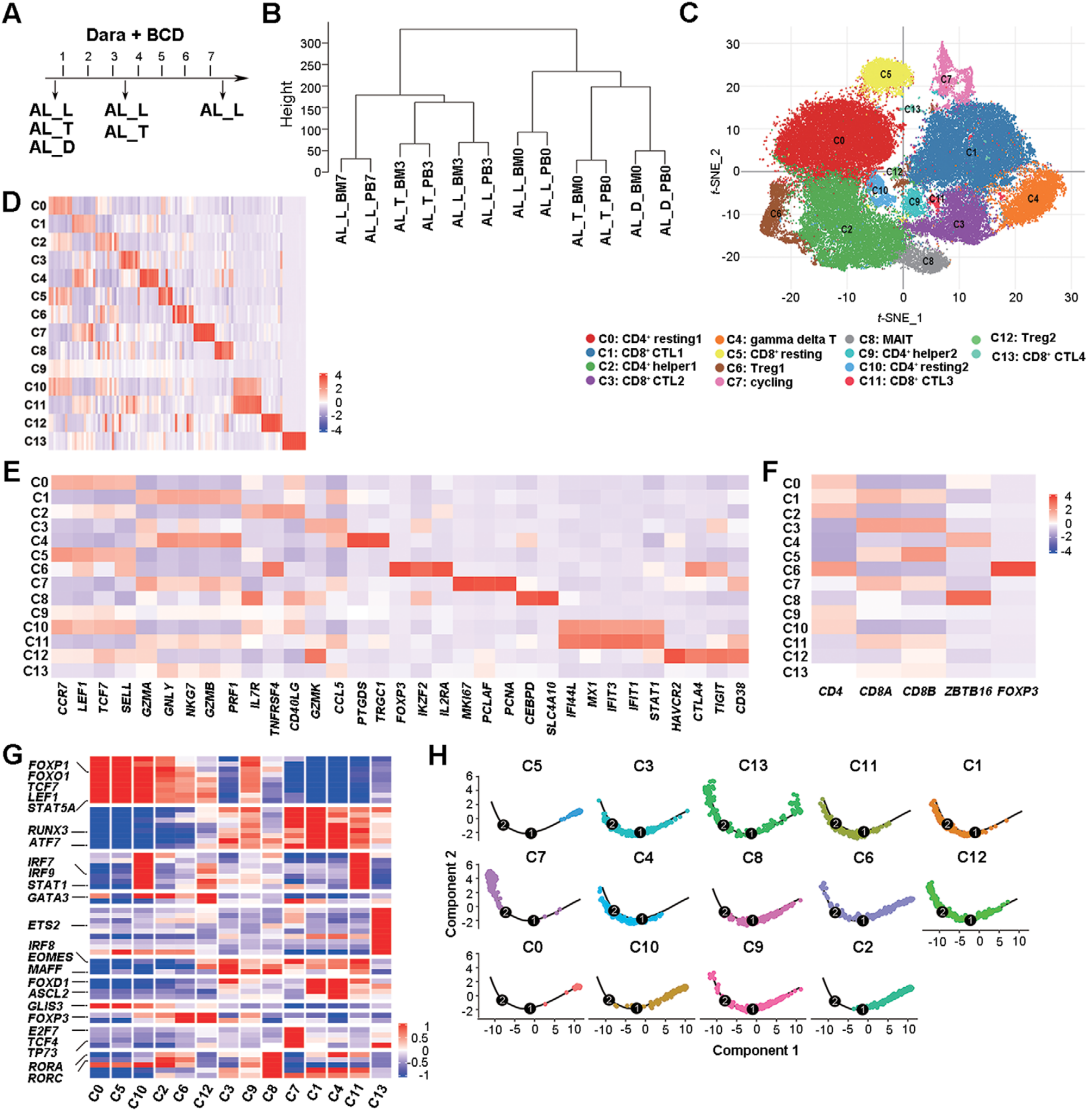
T cell composition in the BM and peripheral blood (PB) of patients with AL amyloidosis. (A) Schematic of the sample collection in AL patients receiving Dara‐BCD regimen. BM aspirates and PB samples were taken at the same time. (B) Pseudo‐bulk analysis and clustering of individual PB and BM T cell samples collected before and after Dara‐BCD. (C) *t‐*SNE visualization of PB and BM T cell clusters. (D) Heatmap showing the row‐scaled expression of the 10 highest DEGs (Bonferroni‐corrected *p* values < .05; student's *t*‐test) per cluster. € Heatmap showing the row‐scaled expression of selected signature genes for each cluster. (F) Heatmap showing the row‐scaled expression of *CD4*, *CD8A*, *CD8B*, *ZBTB16* and *FOXP3*. (G) Heatmap of the cell type‐specific TFs of each cluster, with mean area under the curve scores, are shown. (H): Monocle trajectories of T cells coloured by cluster identity

To investigate T cell alterations in AL amyloidosis, CD3^+^ T cells purified from blood and BM of AL patients before and after Dara‐BCD (Figure 1A and Supplemental Figure S1A) were applied to scRNA‐seq. Three patients with Mayo2012 stage I and III were involved in sample collection before treatment (Supplemental Table S1). Due to the death in one patient (AL_D) and failure of collecting samples from the other patient during the COVID‐19 pandemic (AL_T), two of the three patients (AL_L and AL_T) had samples sequenced after receiving three cycles and one patient (AL_L) after receiving seven cycles of treatment. Notably, AL_T and AL_L both reached complete remission (CR) at the haematological level and partial remission (PR) at a renal level within three cycles of Dara‐BCD.

We first performed pseudo‐bulk expression‐based hierarchical clustering to examine the overall transcriptome pattern across the samples. As shown in Figure 1B, the transcriptomic features of T cells between blood and BM were similar within the same patient at the same time point. Among different patients, T cells before treatment were clustered together and were separate from those after treatment. When BM T cells were analysed alone, the samples before and after treatment were again separate (Supplemental Figure S1B). It indicates that Dara‐BCD in AL patients leads to significant changes in T cells at the transcriptome level.

All the cells from 12 samples were then integrated together and clustered. We used cluster‐specific biomarkers to resolve T cell subsets and functional states into 14 transcriptionally distinct clusters (Figure 1C–F and Supplemental Table S2). The transcription factors (TFs) with high activity within each cluster were revealed by TF regulatory network analysis^29^ (Figure 1G). The clusters with resting T cell features (*LEF1*, *TCF7*), corresponding to naïve or central memory T cells, included C0 (*CD4* ^+^), C10 (*CD4* ^+^, with elevated interferon (IFN)‐responsive genes) and C5 (*CD8A* ^+^ *CD8B* ^+^). We also observed a cluster (C7) of cycling T cells (*MKI67* ^+^) that contained mostly CD8^+^ but also CD4^+^ T cells and four clusters of CD8^+^ cytotoxic T (CTL1‐4) cells expressing different combinations or levels of cytotoxicity‐related genes (C1, C3, C11 and C13). The rest of CD4^+^ T cells were divided into two clusters (C6 and C12) of Treg cells with high *FOXP3* expression and/or activity and two clusters of T helper cells (C2 and C9). We also identified two *ZBTB16^+^NCR3* ^+^ innate‐like T cell clusters, one (C4) with *TRGC1* (TCRγ) and gene signatures similar to CTLs in cluster C1, the other (C8) expressing *SLC4A10* and *RORC*, similar to mucosa‐associated invariant T cells (MAIT) reported by Yao et al.^30^ The proportions of these clusters revealed a degree of heterogeneity among patients, with AL_T having more cytotoxic T cells (C1 and C4) whereas AL_D and AL_L having more T helper cells (C2) (Supplemental Figure S1C‐D). The pseudo‐time analysis further revealed a U‐shaped trajectory path with resting T cell clusters (C0, C5 and C10) at the right, end while the cycling T cells (C7) and cells with high levels of cytolytic gene expression (C1 and C4) at the left end or side of the path (Figure 1H), suggesting that the subsets of CTL1 (C1) and γδ T cells (C4) had more effector cells at terminally differentiated status (T_EMRA_).

**HIGHLIGHTS**

1. Daratumumab with cyclophosphamide, bortezomib, and dexamethasone (Dara‐BCD) treatment leads to an expansion, activation, and type I cytokine upregulation in T cells in AL.
2. CD8^+^ bone marrow T_RM_ cells in AL patients at diagnosis express high levels of inhibitory molecules.
3. CD8^+^ T_RM_ cells downregulate their suppressive molecules and upregulate *IFNG* expression after Dara‐BCD treatment.

### 2.2 Dara‐BCD leads to significant alterations in CD8^+^ T cell subsets

**FIGURE 2:**
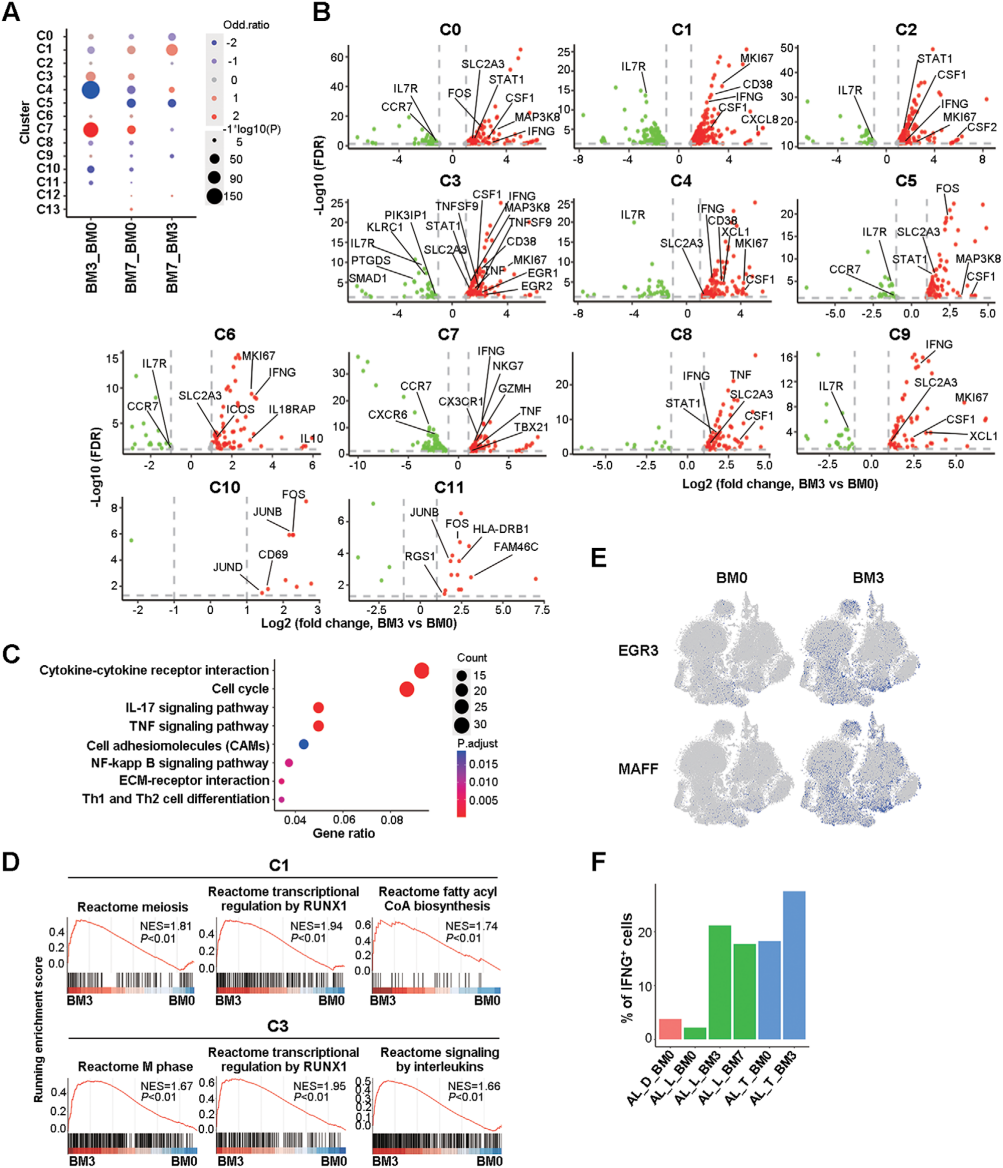
Enhanced T cell activation and expansion in the BM of AL patients receiving Dara‐BCD. (A) Alterations of BM T cell subsets before (BM0) and after three or seven cycles of Dara‐BCD (BM3 and BM7). Each dot represents a cluster in a group of comparison with the dot size representing –1*log10 (*p* value) and dot colour representing cell ratio changes. (B) Volcano plot showing DEGs in each cluster obtained from BM3 and BM0 comparison. The dot lines show a twofold cutoff. (C) KEGG analysis of DEGs obtained from BM3 and BM0 comparison. Selected KEGG terms with hypergeometric test with *p* values < .05 are shown and coloured by *p* adjust values. (D) GSEA analysis of C1 and C3 clusters between BM3 and BM0 samples. (E) *t*‐SNE shows cells coloured by state identity (pySCENIC approach). The identities are inferred by the binary activities of the TF regulons, which show transcription factors such as EGR3 and MAFF as central hubs governing the elevated activation status of T cells in BM3 samples. The activities of TFs– were quantified using pySCENIC (version 0.10.4) aucell module (blue for 1 and grey for 0). (F) Comparison of the percentages of *IFNG* positive T cells in the BM of individual patients before and after treatment

To investigate the impact of Dara‐BCD on BM T cells, we first compared the samples at the cluster level before (BM0) and after 3 (BM3) or 7 (BM7) cycles of treatment. BM3 samples showed a significant increase in cycling T cells (C7) and CTL2 (C3) and a decrease in γδ T cells (C4) relative to BM0 samples (Figure 2A). The additional cycles of treatment (BM7) did not further increase T cells in C7 and C3 subsets. Instead, BM7 had significantly more cells in CTL1 (C1), mildly increased cells in C4 and a clear decline in the resting C5 cluster (Figure 2A). CD4^+^ T cell subsets did not alter much during the treatment, with the exception of a mild decrease in C10 (BM3) and C0 (BM7) (Figure 2A). Different from Dara‐treated MM patients with a reduction in CD38^+^ Treg cells,^24^, ^25^, ^26^ neither BM3 nor BM7 samples showed a decline in the proportion of Treg clusters C6 and C12, even though C12 expressed the highest level of *CD38* transcripts (Figures 1E and 2A). We further observed an upregulation of *CD38* transcription in clusters C1, C3 and C4 in BM3/7 samples, suggesting that these post‐treatment samples had cytotoxic T cells with enhanced activation status (Figure 2B and Supplemental Figure S2A). Collectively, these data demonstrate that Dara‐BCD preferentially promotes CD8^+^ T cell expansion and activation, with the most prominent alterations occurring after three cycles of treatment, being consistent with the dramatic decline of free light chain level during this period (Supplemental Table S1).

### 2.3 Dara‐BCD promotes T cell activation and type I cytokine expression at the transcriptome level

We next compared BM T cells at the differentially expressed gene (DEG) level before and after treatment. As shown in Supplemental Figure S2B, almost all the clusters in BM3 samples had more upregulated than downregulated genes when compared to BM0, with a total of 561 genes upregulated while 195 downregulated (Supplemental Figure S2B). In contrast, BM7 samples showed similar numbers of up‐ and downregulated genes when compared to BM0 but more downregulated genes (with a total of 153 downregulated vs. 44 upregulated genes) when compared to BM3 samples.

We performed the Kyoto Encyclopedia of Genes and Genomes (KEGG) analysisand found that cell cycle, cytokine–cytokine receptor interaction, NF‐κB signalling pathway, Th1 and Th2 differentiation were significantly enriched in DEGs between BM0 and BM3 samples or DEGs within specific cell clusters (Figure 2C and Supplemental Figure S2C). The gene set enrichment analysis (GSEA) analysis of the BM0 and BM3 samples further revealed a significant enrichment of cell cycle–related genes and genes regulated by RUNX1 in CTL clusters C1 and C3 based on Reactome Pathway Database^31^ (Figure 2D). The genes related to interleukin signalling and fatty acyl CoA biosynthesis were also enriched in clusters C3 and C1, respectively (Figure 2D). In addition, the activities of TF such as EGR3 and MAFF were clearly elevated in BM3 when compared to BM0 (Figure 2E). As shown in Figure 2B, most of the clusters in BM3 upregulated *MKI67*, *SLC2A3* (GLUT3) and downregulated *IL7R* and/or *CCR7* expression. These data indicate that three cycles of Dara‐BCD in AL patients lead to substantial changes in the activation, proliferation and metabolism in BM T cells, in particular, CD8^+^ CTLs.

The BM3 cells further revealed a significant upregulation of type I cytokines including *IFNG*, *CSF1*, *TNF*, *CSF2*, *CXCL8* and *XCL1* in CD4^+^, CD8^+^ and innate‐like T cell clusters (Figure 2B). When individual patients were examined, the overall frequency of *IFNG* ^+^ cells was consistently upregulated in the post‐treated samples (Figure 2F). We did not observe a significant increase in the expression of cytolytic genes. As IFN‐γ and IFN‐γ‐producing T cells have been shown to play critical roles in the control of MM as well as a variety of other types of tumors,^32^, ^33^, ^34^ these results indicate that Dara‐BCD promotes anti‐tumour immune response by upregulating type I cytokine expression in T cells.

### 2.4 Dara‐BCD promotes the activation of CD8^+^ BM resident memory T cells

**FIGURE 3:**
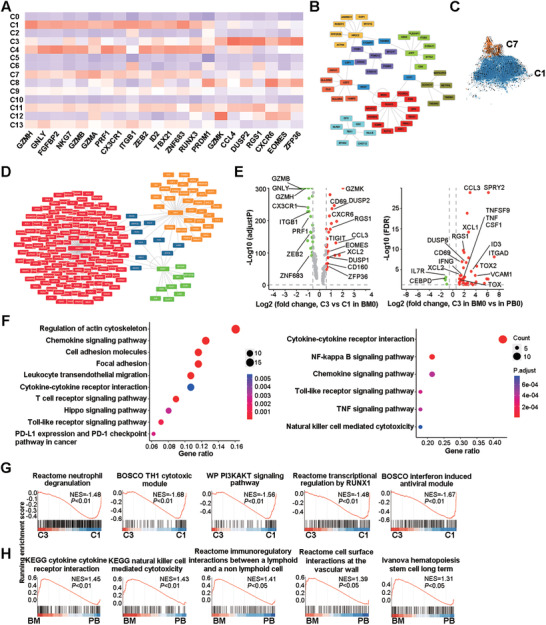
Comparison of CD8^+^ T_EMRA_ and T_RM_ cells in the BM of AL amyloidosis. (A) Heatmap showing the row‐scaled expression of signature genes for C1 (T_EMRA_) and C3 (T_RM_) cells. (B) Gene regulatory network analysis using pySCENIC (version 0.10.4) identifies critical nodes (*RUNX3, USF2, NR2C2, PSMD12, ATF7, TBX1*, *and* *ZCCHC17*) in cluster C1. The predicted TFs and their target genes are shown. (C) RNA velocities inferred by scVelo are visualized on the pre‐defined *t*‐SNE plot from Seurat coordinates of clusters C1 and C7. (D) Critical nodes (*EOMES, MAFF* and *IRF8*) in cluster C3 are identified by gene regulatory network analysis (pySCENIC). The predicted TFs and their target genes are shown. (E) Volcano plot showing DEGs (Wilcox test *p* values < .01) in the comparison of clusters C3 and C1 before DARA‐based therapy (left panel) and DEGs in the comparison of BM‐ and peripheral blood‐derived C3 cells before DARA‐based therapy (right panel). The dot lines show a 1.5‐fold cutoff. (F) KEGG analysis of DEGs in the comparison of clusters C3 and C1 in BM0 samples (left panel) and DEGs in the comparison of BM0‐ and PB0‐derived C3. (G) GSEA analysis of BM0‐derived C3 and C1 cell comparison. (H) GSEA analysis of DEGs in the comparison of BM0‐ and PB0‐derived C3 cells

As Dara‐BCD led to more prominent alterations in CD8^+^ than CD4^+^ T cells at the cluster level, we examined the two major CTL clusters C1 and C3 in more details. The C1 cluster had the transcriptomic features of T_EMRA_ ^35^ with high expression of TFs such as *ZEB2*, *TBX21*, *PRDM1*, chemokine receptor *CX3CR1*, NKRs (*KLRG1*) and a series of cytotoxicity‐related genes (*GZMH*, *GZMB*, *GNLY*, *PRF1*) (Figure 3A). The activity of TFs such as RUNX3 was specifically high in C1 (Figures 1G and 3B). After three cycles of Dara‐BCD, an upregulation of *IFNG*, *CSF1* and *CXCL8* and downregulation of *CXCR6* was observed in C1 cells (Figure 2B). Notably, the cycling cells in C7 showed a similar profile of TF activity as those in C1 (Figure 1G) and a trend of differentiation towards C1 by RNA velocity analysis (Figure 3C). In addition, C7 cells in BM3 samples upregulated C1 marker genes such as *TBX21*, *GZMH*, *NKG7* and *CX3CR1* (Figure 2B). Together with a late increase in C1 cell proportion in BM7 samples, these data demonstrate that repeated cycles of Dara‐BCD induce proliferation and differentiation of CD8^+^ T cells into T_EMRA_ cells with high expression of type I cytokines and cytolytic molecules.

The C3 cluster, however, had the gene signatures being reminiscent of memory T cells, with high levels of TF activity (EOMES, IRF8 and MAFF) and expression (*EOMES*, *ZFP36*, *NR4A2*), high expression of *GZMK* and *CXCR6* (Figures 1, 3 and 3D–3E).^36^ We further found that C3 cells expressed high levels of non‐lymphoid tissue‐retaining molecules *CD69* and *RGS1*.^37^ When compared to the smaller proportion of C3 in the blood (5.19 ± 0.73%), the larger proportion (7.16 ± 1.29%) of C3 cells in BM showed higher expression of *RGS1*, *VCAM1* and its receptor *ITGAD* (Figure 3E). Together with the KEGG (Figure 3F) and GSEA (Figures 3G–H) analysis, it demonstrates that cluster C3 was enriched with tissue‐resident T cells (T_RM_). Notably, *ZFP36* was found to suppress T cell proliferation^38^ and *NR4A2* had a positive correlation with *PDCD1* and *HAVCR2* expression in human melanoma infiltrating cells.^39^ Compared to the circulating C3 or BM‐derived C1 cells, BM‐derived C3 T cells expressed higher levels of a group of negative regulators, including *TOX*, *TOX2*,^40^ *SPRY2*, *KLRC2*, *DUSP6*, *DUSP2*, *DUSP1*, *TIGIT*, and *CD160*.^41^ We further extracted scRNA‐seq data (GSE124310)^36^ from the BM of healthy donors and found the presence of similar *CD69* ^+^ *RGS1* ^+^ *GZMK* ^+^ *ZFP36* ^+^ T_RM_ cell cluster (Supplemental Figure S3A‐B). The expression of the above negative regulators, however, was not seen. The ratio of BM T_RM_ and T_EMRA_ was also much higher in healthy donors (5.48 ± 4.83) than AL patients (0.41 ± 0.26), suggesting an expansion of T_EMRA_ cells in the latter group. These data together demonstrate that patients with AL amyloidosis have a population of CD8^+^ BM T_RM_ cells with impaired anti‐tumour activity.

After three cycles of treatment, the proportion of cluster C3 in the BM was increased (Figure 2A). Some of the negative regulators such as *SMAD1*,^42^ *PIK3IP1*,^43^ *PTGDS*,^44^ *PLAC8*,^45^ and *KLRC1* were significantly downregulated, while *TNFSF9*, *CD70*, *EGR1*, *EGR2, MAP3K8, STAT1*, and *SLC2A3* were significantly upregulated in C3 cells (Figure 2B), indicating that Dara‐BCD induces an expansion and activation of BM T_RM_ cells.

### 2.5 TCR analysis reveals T cell expansion in the BM

**FIGURE 4:**
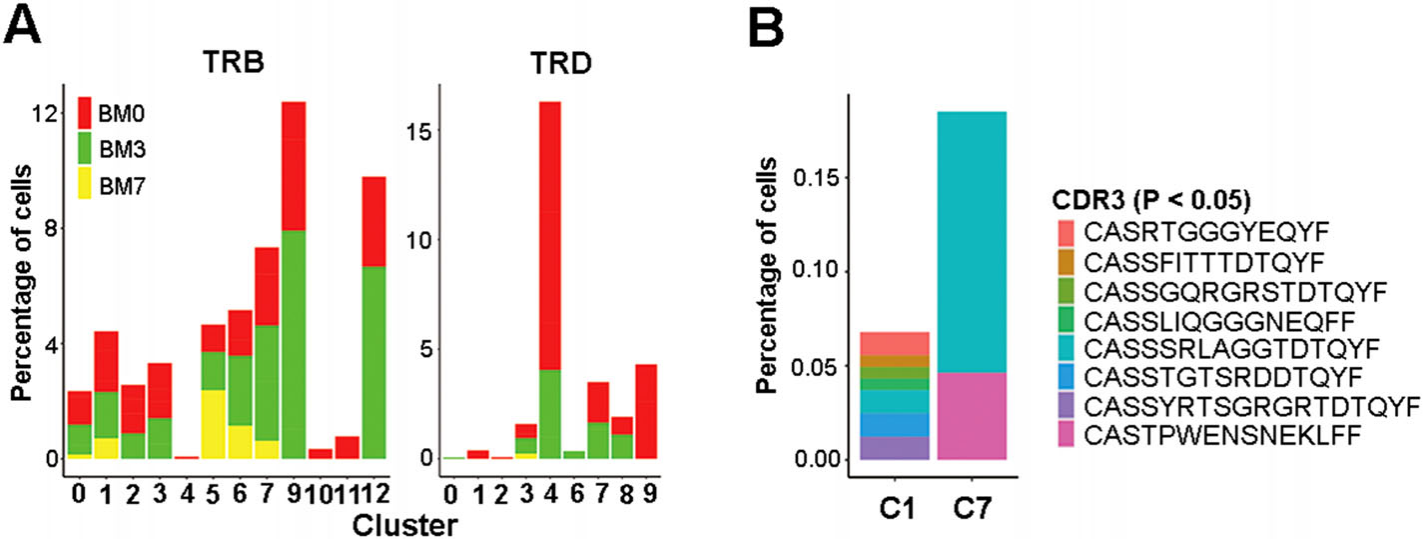
Clonal alterations and expansion of T cells (defined by TCR) in patients receiving Dara‐BCD. (A) TCR identification of T cells within each cell cluster. (B) Percentage distribution of CDR3 clonotypes in clusters C1 and C7 that were significantly increased in post‐treatment samples relative to the ones before treatment (*p* < .05, Fisher exact test)

The sequencing of T cells was from 3′‐end, making the identification of TCR sequences distal to 3′ of mRNAs very difficult. However, we applied the MiXCR algorithm to identify paired V/J segments and CDR3 sequences of TCR genes for as many T cells as possible. As shown in Figure 4A, both αβ and γδ TCRs could be identified in the samples. In particular, the *TRGC1* ^+^ cluster C4 showed enrichment of Vγ9Vδ2 TCRs, confirming that cells in this cluster were mostly γδ T cells. The identification of CDR3 is limited from the 3′end; however, the percentages of CDR3 in clusters C1 and C7 in the comparison of BM3 and BM0 samples clearly support T cell expansion (Figure 4B).

### 2.6 T‐plasma cell interaction analysis reveals alterations after Dara‐BCD

**FIGURE 5:**
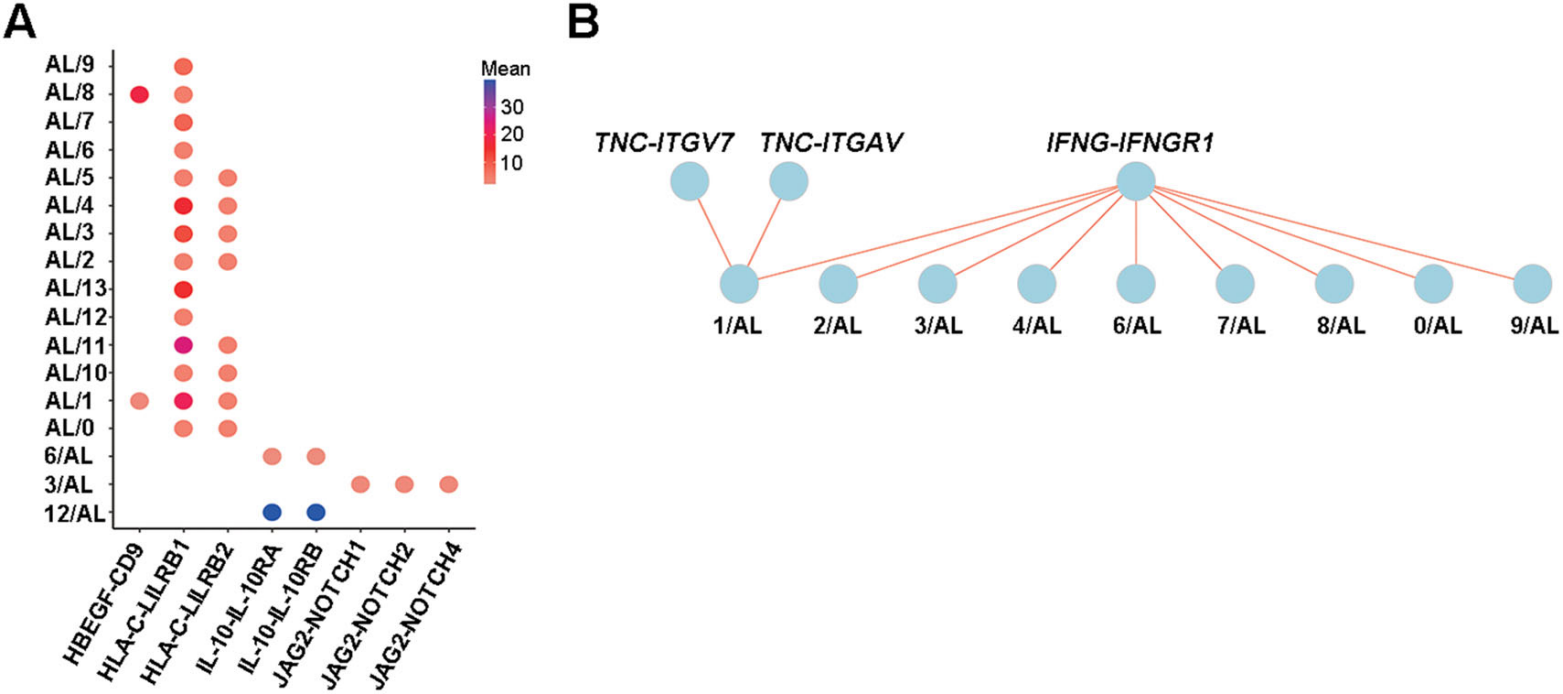
Interaction analysis of T cells and clonal plasma cells in patients with AL amyloidosis. (A) Bubble plot shows the mean expression of ligand and receptor pairs co‐expressed between PCs (AL) and T cell clusters. (B) Biopartie graph shows the ligand and receptor pair interaction between different clusters, the red line shows an enhanced communication

To study the anti‐tumour response in T cells, the potential communication between T cells and aberrant PCs was analysed. We previously reported overexpression of MHC class I molecules in AL‐derived PCs using the scRNA‐seq data from the GEO database (GSE117156).^46^ The same PC data were applied in the analysis of T‐PC interaction. In BM0 samples, *HLA‐C* from PCs may interact with inhibitory molecules *LILRB1* or *LILRB2* expressed in all T cell clusters. We also identified pairs of *IL‐10* and *IL‐10RA/B*, *JAG2* and *NOTCH1/2/4*, *HBEGF* and *CD9* that may suppress T cell response or promote tumour cell growth or drug resistance (Figure 5A).^47^, ^48^, ^49^ When DEGs from BM3 and BM0 comparison were used, a high level of *IFNG* expressed by T cells was found to interact with *IFNGR1* expressed by PCs. We additionally found TNC‐ITGA7/V pairs between C1 cells and PCs, suggesting an enhanced adhesion of T_EMRA_ with aberrant PCs. Together, the cell interaction analysis further supports that T cell function within the vicinity of clonal PCs is impaired whereas Dara‐BCD promotes T cell anti‐tumour response by upregulating IFN‐γ.

### 2.7 Dara‐BCD leads to similar T cell alterations in blood

**FIGURE 6:**
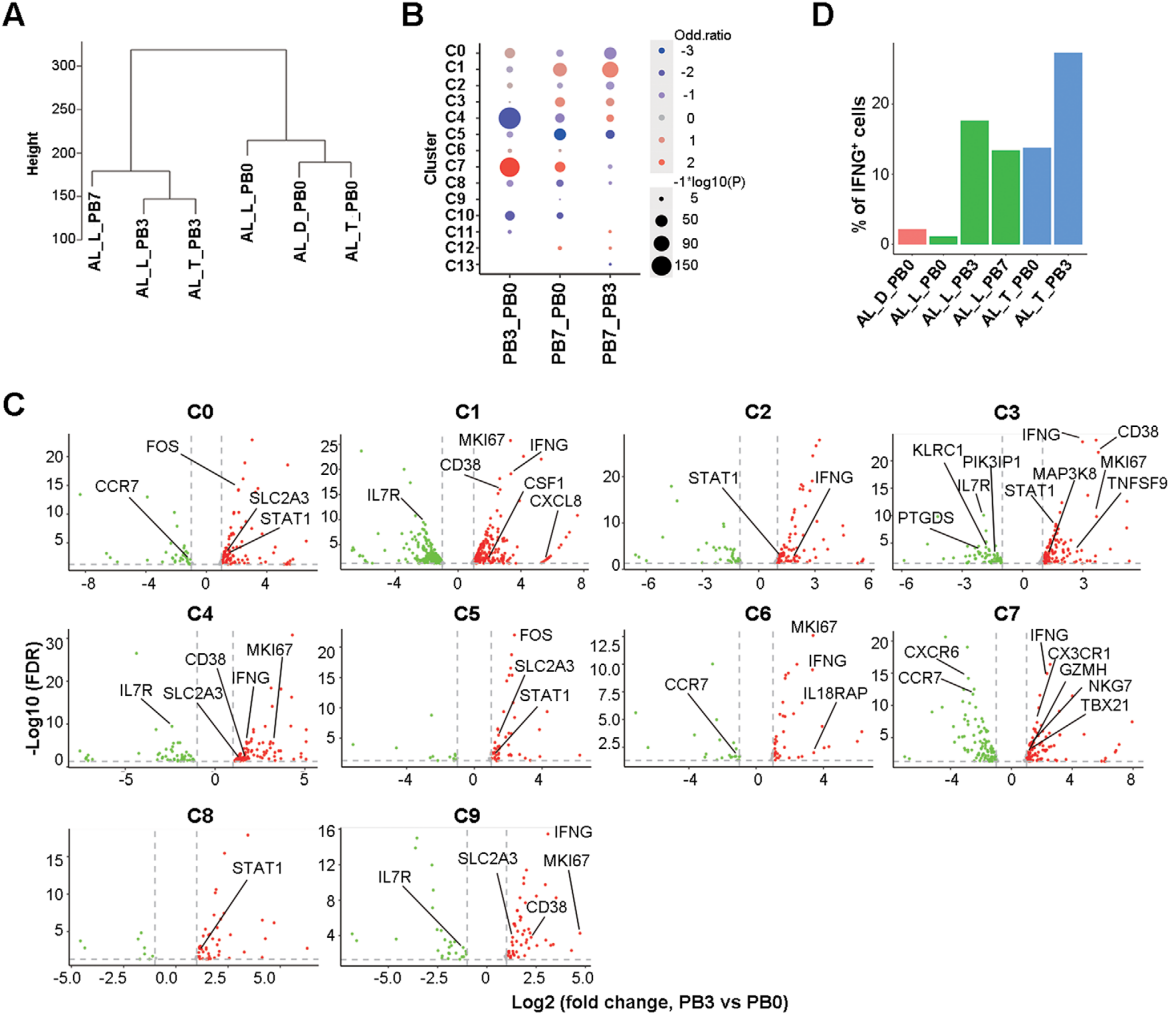
Similar alterations in BM T cells and circulating T cells before and after Dara‐BCD. (A) Pseudo‐bulk analysis and clustering of individual circulating T cell samples collected before and after Dara‐BCD. (B) Alterations of the circulating T cell subset proportion before and after Dara‐BCD. Each dot represents a cluster with dot size representing –1*log10 (*p* value) and dot colour representing log odd ratio of cluster size between different therapeutic time points. (C) Volcano plot showing DEGs in each cluster after and before Dara‐BCD. The dot lines show twofold cutoff. (D) Percentage of *IFNG* positive T cells in the circulating T cells in individual patients before and after treatment

We next determined whether circulating T cell characteristics can be used to monitor T cell response following Dara‐BCD as similar features were found between circulating and BM T cells by pseudo‐bulk analysis (Figure 1B). As shown in Supplemental Figure 3C, very few DEGs were seen in each cluster (except C3) when circulating and BM T cells were compared. We further found that circulating T cells before treatment were clustered together and were separate from those after treatment (Figure 6A), and the proportions of cycling T cells (C7) in PB3 and T_EMRA_ (C1) in PB7 were significantly increased (Figure 6B), both agreeing well with the alterations in BM samples. Notably, the cluster C3 in blood did not expand after three cycles of treatment, further supporting that C3 cells in BM are T_RM_ cells. The pattern of up‐ and downregulated genes within each cluster in the comparison of circulating T cells before and after treatment was similar to BM T cells (Figure 6C and Supplemental Figure S3D). Specifically, we also found an upregulation of *IFNG* transcription in circulating T cells after three or seven cycles of Dara‐BCD (Figure 6D). Together, these data indicate that the overall changes in circulating and BM T cells induced by Dara‐BCD were similar, suggesting that tracking of anti‐tumour immunity in patients with AL could be realized by a less invasive method of taking peripheral blood samples.

## 3 DISCUSSION

High expression of CD38 in abnormal PC clones and recent reports on the safety and response rate of Dara have made anti‐CD38 antibody‐based therapy a promising approach in AL amyloidosis.^50^ In the analysis of three newly diagnosed AL patients before treatment and two of them after Dara‐BCD treatment, we found significant T cell activation, expansion and upregulation of type I cytokine expression post‐treatment, with the most prominent changes occurring in CD8^+^ T cells. Different from MM patients that had Dara‐induced CD38^+^ Treg cell depletion,^25^, ^26^, ^51^ we did not observe an alteration in CD38^hi^ (C12) or CD38^lo^ (C6) Treg cell proportion. The transcription of *CD38* was even upregulated in CTLs after treatment. Whether this elevation of *CD38* expression is an indicator of T cell activation^27^, ^52^ or works as a regulatory molecule^53^, ^54^, ^55^, ^56^ in AL awaits further investigation.

Cytotoxic T cells and Tc1 immunity are central for tumour control. We found CD8^+^ T cell activation and expansion after Dara‐BCD. In particular, the expression of type I cytokines, including *IFNG*, was significantly elevated in CTLs after treatment. The T‐PC interaction analysis further revealed an upregulation of *IFNG*‐*IFNGR1* co‐expressing pair between the two cell types. IFN‐γ has a direct antitumor effect via inhibiting proliferation or inducing apoptosis/necroptosis of tumour cells and an indirect antitumor effect via changing the capabilities of T and non‐T cells against tumors.^57^, ^58^ The impact of IFN‐γ on aberrant PCs in AL amyloidosis is unclear. In MM patients, however, a high level of *IFNG* has been shown to be independently correlated with patient survival;^59^ increased frequency of IFN‐γ^+^TNF^+^ CD8^+^ BM T cells was associated with the profound immune‐driven control of myeloma in a BM transplantation model.^32^ Deficiency in *Ifng* had accelerated tumour growth and lethality in a mouse model.^60^ In addition to *IFNG*, we also observed an upregulation of *CSF1* (M‐CSF) and *CXCL8* in CTLs after treatment. CXCL8 is a potent neutrophil chemotactic factor while M‐CSF, together with IFN‐γ, promotes the activation of tumouricidal M1 macrophages.^58^, ^61^ Collectively, the upregulation of these cytokines in CTLs indicate that Dara‐BCD promotes Tc1‐based anti‐tumour immune response in AL amyloidosis.

Memory T cells are heterogeneous, and T_RM_ cells, in comparison to other memory cell subsets, preferentially provide tissue‐specific surveillance and protection. The BM is a ‘reservoir’ for memory T cells, but BM T_RM_ cells are less well studied.^62^, ^63^, ^64^ In the BM of AL patients, we found a cluster (C3) of CD8^+^ *GZMK* ^+^T_RM_ cells with increased expression of *EOMES* TF target genes, high expression of genes associated with non‐lymphoid tissue residence (*CD69*, *CXCR6*, *RGS1*, *VCAM1* and its receptor *ITGAD*) and low expression of T_EMRA_‐related genes. In the BM of MGUS and MM patients, a somewhat similar subset was reported by Vuckovic, et al. (Eomes^hi^CD69^+^ T_TE_ cells)^65^ and Bailur et al. (*TCF7* ^hi^ T_RM_ cells).^63^ Notably, BM T_RM_ cells in newly diagnosed AL patients expressed high levels of negative regulators such as *TOX*,^40^ *SPRY2*, *DUSPs* and *TIGIT*,^41^ suggesting their impaired anti‐tumour function against the aberrant PCs. The potential T‐PC communication by the expression of the JAG2/NOTCH pair may further promote the survival and proliferation of clonotypic PCs (Figure 5A).^49^ Dara‐BCD induced the upregulation of proliferation‐ and activation‐related genes as well as type I cytokines in T cells. It also downregulated the inhibitory molecules including *PIK3IP1*, *KLRC1*, *PTGDS* and *SMAD1*, further promoting T cell activation and differentiation. Collectively, these data indicate that the presence, characteristics and expansion of CD8^+^ BM T_RM_ cells are likely essential in the control of tumour cells in AL amyloidosis. Relieving the inhibitory brakes in BM T_RM_ cells may be critical in the design and/or monitor of the immuno‐therapy against this disease.

The current study has some limitations. First, the sample size was small with three patients included before treatment and only two of them after three cycles of Dara‐BCD treatment due to one death (AL_D) right after the first cycle of treatment. The COVID‐19 pandemic also made it difficult to recruit patients in a timely manner and eventually leading to the missing of the sample collection by the third patient (AL_T) after seven cycles of treatment. To avoid introducing bias into the study, we analysed the data twice by including (Figures 1, 2, 3, 4, 5, 6) and excluding (Supplemental Figure S4) the scRNA‐seq data obtained from AL_D. Similar results were obtained (Figures 1, 2, 3 and Supplemental Figure S4), suggesting that the inclusion of AL_D in the pretreatment cohort did not affect the results. The scRNA‐seq data after seven cycles of treatment were obtained from only one patient, but their close relationship and similarity with the data after three cycles of treatment (Figure 1B and supplemental Figure S1B and 2B) allowed us to focus our comparison efforts more on the data before and after three cycles of treatment. However, further investigations with a larger sample size and other immune cells included will be important to confirm the results and extend our understanding to a full picture of the immune microenvironment of AL before and after treatment. Second, we only examined the patients receiving a combination of Dara and BCD. The impact of BCD alone or bortezomib‐based regimens on T cells has been investigated in patients with MM but not AL. In patients with MM, both bortezomib and Dara treatments led to a significant reduction in NK cells.^28^ However, different from Dara‐treated MM patients, bortezomib‐treated ones showed a significant decrease in CD3^+^ T cells, CD4^+^ T cells and an increase in CD8^+^ T cells.^28^ The frequencies of Treg cells were not altered.^66^ The functions of T cells were likely impaired due to the pro‐apoptotic effect of bortezomib on dendritic cells.^67^, ^68^ Although the impact of BCD treatment alone on T cells is yet to be determined in AL patients, the similar risk of cytomegalovirus reactivation in bortezomib‐treated MM and AL patients suggests that bortezomib may induce similar T cell changes in AL patients.^69^, ^70^

In addition, in the patients who got an early and deep response,^71^ the samples after only one cycle of Dara‐based therapy should be collected to study an early T cell immune response. Prolonged IFNG signalling is well known to induce the expression of immune checkpoint molecules such as PD‐L1, thus resulting in tumour immunoevasion.^72^ Whether this is true in Dara‐based therapy is awaiting further investigation. We also noticed a significant reduction in the proportion of cluster C4 in samples after treatment (Figures 2A and 6B). C4 was enriched with Vγ9Vδ2 T cells and showed similar T_EMRA_ gene signatures as C1 (Figure 1E). Whether these Vγ9Vδ2 T_EMRA_ cells in AL patients express a high level of CD38 that could be depleted in the treatment with anti‐CD38 antibody and whether these cells are dysfunctional like those in chronic HCV infection^73^ will be an important question to investigate.

Taken together, the T cell transcriptome at a single‐cell level revealed an elevated cell activation, expansion, and IFN‐γ‐based anti‐tumour immunity in AL patients receiving repeated cycles of Dara ‐based therapy.

## 4 METHODS

### 4.1 Basic information for patients with AL amyloidosis

We collected mononuclear cells from the blood and BM samples of patients with newly diagnosed λ‐type AL amyloidosis before and after a combination of D‐BCD (aged 42–66 years, Mayo2012 staged as I (*n* = 1), III (*n* = 2; Supplemental Table S1). The frequency of aberrant PC in the BM was ranged from 5.5% to 27.5%. Among them, AL_D died of an infection after the first cycle of treatment, thus lacking samples post‐treatment. AL_L had samples before (PB0, BM0) and after three cycles (PB3, BM3) and seven cycles (PB7, BM7) of treatments while AL_T had samples before and after three cycles of treatments but no sample collection after seven cycles of treatment due to COVID‐19 pandemic (Supplemental Table S1).

### 4.2 Sample preparation

The mononuclear cells were prepared, and CD3^+^ cells were enriched with magnetic beads (Miltenyi Biotec B.V. & Co. KG, Germany) and were sent to CapitalBio Technology Inc. for 10× Genomics single‐cell RNA sequencing (scRNA‐seq). Specifically, the single‐cell gel bead in emulsion was prepared by loading cellular suspensions on a single‐cell 3′ chip together with the single‐cell 3′ gel beads. scRNA‐seq libraries were prepared using the chromium single‐cell 3′ library kit v.3 (10× Genomics). Reverse transcription, amplification and sequencing were performed using an S1000™ Touch Thermal Cycler (Bio‐Rad) and Illumina Novaseq6000 platform, respectively.

### 4.3 Data processing

The R package Seurat (version 3)^74^ was used for data scaling, transformation, clustering, dimensionality reduction, differential expression analysis and visualization.

The Cell Ranger Single‐Cell Software Suite v.3.0.2 was used for sample demultiplexing, alignment (GRCh38 human reference genome with STAR^75^), barcode processing and unique molecular identifier counting (see Supplemental Methods). A total of 68,166 cells from 12 specimens passed the quality control, with 3561 to 7553 cells in each sample. We performed Seurat (v3.0, R package) *k*‐nearest neighbours clustering with resolution 0.5 on the first 50 principal components. Fisher's exact test (KEGG pathway database) and GSEA algorithm (MSigDB c2.all.v7.2 database)^76^ were used for KEGG enrichment and GSEA analysis, respectively. All the results were visualized using the R package.

Monocle2 (R package) was used to build pseudo‐time trajectories and scVelo (0.2.2) software was used for RNA velocity analysis. The velocities were visualized on the pre‐defined *t*‐SNE plot from Seurat coordinates. The pySCENIC (0.10.4) software was used for TFs regulatory network analysis. Cell–cell interaction analysis was based on the expression of pairs of receptors and ligands.^77^ The interaction score of each ligand–receptor pair was defined as the product of the average expression levels of the ligand in one cell type and the corresponding receptor in the other cell type.

The transcriptome of each sample was aggregated to pseudo‐bulk expression data to check the overall transcriptomes across samples. The pseudo‐bulk gene expression data were clustered using the hierarchical clustering algorithm based on the Euclidean distance matrix.

TCR sequences were computed using the MiXCR tool v3.0.9,^78^ reference germline V, D, J, and C gene sequences were downloaded from the IMGT database.^79^

## CONFLICT OF INTEREST

The authors have declared that no competing interests exist.

## STUDY APPROVAL

All the blood and BM samples were collected after informed consent and in compliance with the Declaration of Helsinki under a protocol approved by the Research Ethics Board in Peking University People's Hospital. Informed consent was obtained from all patients for being included in the study.

## Supporting information



SUPPORTING INFORMATION

SUPPORTING INFORMATION

## Data Availability

The raw scRNA‐seq data have been deposited in the Genome Sequence Archive^80^ in National Genomics Data Center^81^, China National Center for Bioinformation/Beijing Institute of Genomics, Chinese Academy of Sciences, under accession number HRA000559 that are publicly accessible at http://bigd.big.ac.cn/gsa‐human. Previously published scRNA‐seq data that were used in this study are available under accession code GSE124310 and GSE117156.
