# Supplementary material for: T cell landscape and dynamics in immunoglobulin light chain amyloidosis before and after daratumumab‐based therapy
Source: Clin Transl Med. 2021 Nov 29;11(11):e582. doi: 10.1002/ctm2.582 (PMC8630449; doi:10.1002/ctm2.582)

Title: T cell landscape and dynamics in immunoglobulin light chain amyloidosis before and after Daratumumab-based therapy

Yujia Wang^1^, Lushuang Xu^1^, Weijia Zhao^1^, Xiaojie Chen^2^, Lei Wen^3^, Wenbing Duan^3^, Xiao-Juan Yu^4, 5, 6, 7^, Fu-De Zhou^4, 5, 6,^ 7, Yang Liu^3^, Jie Hao^1^, Xiaojun Huang^3^, Jin Lu^3, 8^, Qing Ge^1, 9, 10^

**Supplemental Methods**

*Alignment and quality control of sequencing data.*

The Cell Ranger Software Suite (version 3.0.2; 10x Genomics) was used to perform sample de-multiplexing, alignment, barcode processing, and unique molecular identifier (UMI) counting. The sequencing reads were aligned against the GRCh38 human reference genome with STAR ^1^, and count matrices were built from the resulting BAM files.

The quality of cells was then assessed based on three metrics step by step: (1) The number of total UMI counts per cell; (2) The number of detected genes per cell; (3) The proportion of mitochondrial gene counts. The following criteria were then applied to filter low-quality cells: UMI number < 1000, gene number < 200 or > 6,000, proportion of mitochondrial gene > 0.3.

*Integration of cells from different samples*

To remove batch effect, all samples were integrated with harmony ^2^, an algorithm that projects cells into a shared embedding in which cells were grouped by cell types rather than dataset-specific conditions.

*Dimension reduction and visualization*

The filtered gene-cell matrix was first normalized using ‘LogNormalize’ methods in Seurat v.3 with default parameters. The top 2,000 variable genes were then identified using the ‘vst’ method in Seurat FindVariableFeatures function. PCA was performed using the top 2,000 variable genes. The *t*-SNE was performed on the top 25 principal components for visualizing the cells.

*Clustering of cells*

Graph-based clustering was performed on the PCA-reduced data for clustering analysis with Seurat v.3. The resolution was set to 0.5 to obtain a finer result. Briefly, the first 50 PCs of the integrated gene-cell matrix were used to construct a shared nearest-neighbor graph (SNN; FindNeighbors in Seurat) and this SNN was used to cluster the dataset (FindClusters) using a graph-based modularity-optimization algorithm of the Louvain method for community detection.

*Cluster-specific biomarker analysis*

Wilcoxon rank sum test in FindAllMarkers function (with default parameters) in Seurat was used to identify Cluster-specific genes/biomarkers. For each cluster, biomarkers were those highly expressed relative to all other cells. Differentially expressed genes (DEGs) between different sample groups within a cluster were identified using edgeR, a R package, with default parameters. A gene was considered significantly differentially expressed if false discovery rate (FDR) < 0.05.

*KEGG pathway analysis and GSEA analysis*

KEGG pathway analysis were performed with Fisher’s exact test on KEGG pathway database ^3^. GSEA uses kolmogorov-smirnov test to tell whether a given set of genes were significantly enriched or depleted according to fold-changes of gene expression. In this paper, we chose MSigDB c2.all.v7.2 database as gene set input for GSEA analysis.

*Cellular identity analysis*

Cellular identity was determined by comparing cluster-specific biomarkers of each cluster to known cell type-specific genes from previous studies. Cluster annotation was confirmed using the R package SingleR ^4^, which compares the transcriptome of each single cell to reference datasets to determine cellular identity.

*Pseudo-time trajectories*

Analysis of pseudo-time trajectory was performed using R package monocle2 ^5^, ﻿an algorithm defining the transcriptional dynamics of a temporal process. ﻿Monocle revealed switch-like changes in expression of key regulatory factors, sequential waves of gene regulation, and expression of regulator ﻿during a wide array of cellular processes, including differentiation, proliferation and oncogenic transformation.

*Cell-cell interaction analysis*

The cell-cell interaction analysis was based on the expression of pairs of receptors and ligands. The 2, 364 pairs of well-annotated receptors and ligands, including cytokines, chemokines and co-stimulators ^6^ were applied in this study. The interaction score of each ligand-receptor pair was defined as the product of the average expression levels of the ligand in one cell type and the corresponding receptor in the other cell type. Interaction scores between two cell types were defined as sum of all ligand-receptor scores within the two cell types. We defined this ligand-receptor pair as a potential molecular axis mediating interactions between the two cell types when at least one of the ligand-receptor is a marker gene of one cell type or is differentially expressed in a group of compare.

*Pseudo-bulk analysis*

Bulk analysis provides different point of view of alterations at sample level other than at single cell level. Pseudo-bulk sample clustering analysis followed by clustering with the hierarchical clustering algorithm based on correlation coefficients distance matrix (hclust R function).

*RNA velocity analysis*

RNA velocity recapitulates dynamics of T cells differentiation. The scVelo (0.2.2) software was used for RNA velocity analysis. The velocities were visualized on the pre-defined *t*-SNE plot from Seurat coordinates.

*SCENIC analysis*

The pySCENIC (0.10.4) software was used for transcription factors regulatory network analysis, which is faster than SCENIC (Single-Cell Regulatory Network Inference and Clustering).

**Reference for supplemental materials**

1. Dobin A, Davis CA, Schlesinger F, et al. STAR: ultrafast universal RNA-seq aligner. *Bioinformatics*. 2013;29(1):15-21.

2. Korsunsky I, Millard N, Fan J, et al. Fast, sensitive and accurate integration of single-cell data with Harmony. *Nat Methods*. 2019;16(12):1289-1296.

3. Kanehisa M, Goto S. KEGG: kyoto encyclopedia of genes and genomes. *Nucleic Acids Res*. 2000;28(1):27-30.

4. Aran D, Looney AP, Liu L, et al. Reference-based analysis of lung single-cell sequencing reveals a transitional profibrotic macrophage. *Nat Immunol*. 2019;20(2):163-172.

5. Qiu X, Mao Q, Tang Y, et al. Reversed graph embedding resolves complex single-cell trajectories. *Nat Methods*. 2017;14(10):979-982.

6. Ramilowski JA, Goldberg T, Harshbarger J, et al. A draft network of ligand-receptor-mediated multicellular signalling in human. *Nat Commun*. 2015;6:7866.

7. Zavidij O, Haradhvala NJ, Mouhieddine TH, et al. Single-cell RNA sequencing reveals compromised immune microenvironment in precursor stages of multiple myeloma. *Nature Cancer*. 2020.

**Supplemental Tables (see files in EXCEL)**

Supplemental Table 1 Detailed clinical characteristics. Detailed demographic, clinical, and disease treatment and progression data for all patients.

Supplemental Table 2 Differentially expressed genes (top 30 DEGs) across clusters of AL-derived and healthy donor-derived T cells.

Supplemental Table 3 Gene set enrichment analysis by KEGG analysis.

Supplemental Table 4 TCR analysis.

Supplemental Table 5 Cell-cell interaction analysis between T and aberrant plasma cells.

Supplemental Table 6 DEGs between BM and PB samples at the same time point and BM samples at different time points.

**Supplemental Figures and figure legends**

Supplemental Figure 1 Purification and heterogeneous composition of T cells in the BM and peripheral blood of patients with AL amyloidosis. A. Purification of CD3^+^ peripheral blood and BM T cells for scRNA seq. B. Pseudo-bulk analysis and clustering of individual BM T cell samples collected before and after Dara-BCD. C. *t-*SNE visualization of circulating and BM T cell clusters from individual patient before and after Dara-BCD. D. Distribution of T cell clusters in individual patient with AL amyloidosis.


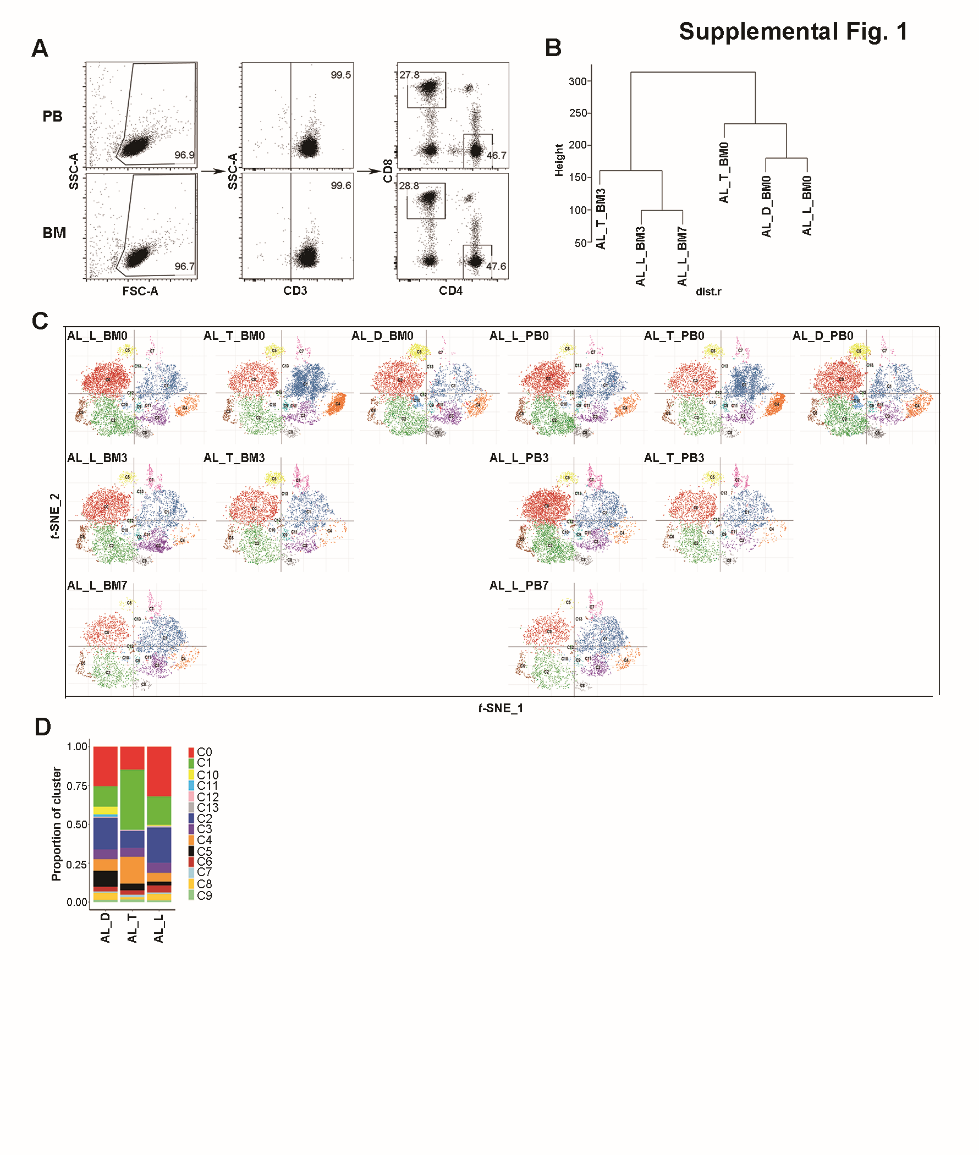


Supplemental Figure 2 Comparison of BM T cells before and after Dara-BCD. A. Volcano plots showing DEGs in clusters C1, C3, and C4 obtained from BM7 and BM0 comparison. The dot line shows 2-fold cutoff. The expression of *CD38* was indicated. B. The numbers of upregulated and down-regulated genes in BM T cells before and after Dara-BCD. C. KEGG analysis of DEGs of indicated clusters obtained from BM3 and BM0 comparison. Selected KEGG terms with hypergeometric test with *P* values < 0.05 are shown and colored by *P* adjust values.


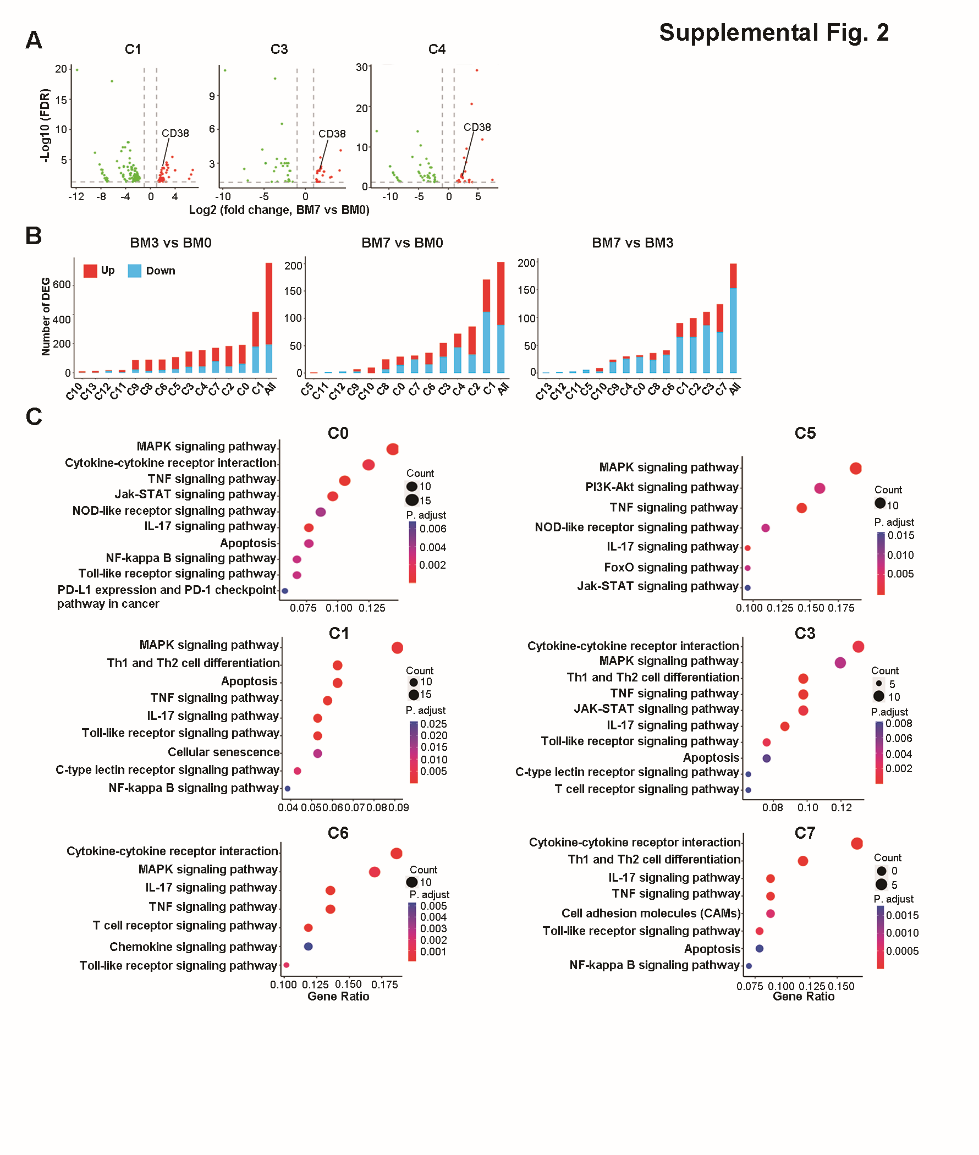


Supplemental Figure 3 T cell composition in the BM of healthy donors and comparison of blood and BM T cells in patients with AL amyloidosis. A. *t-*SNE visualization of T cell clusters from the BM of healthy donors (GSE124310) ^7^. B. Heatmap showing the row-scaled expression of indicated genes in BM T cell clusters from healthy donors. C. Venn diagram showing the number of upregulated (in orange circle) and down-regulated (in blue circle) genes in the comparison of blood and BM T cells in each cluster. The number in the green circle indicates the total number of genes identified in T cells in each cluster (Bonferroni-corrected P values < 0.05; student’s *t*-test). D. The numbers of upregulated and down-regulated genes in PB T cells before and after Dara-BCD.


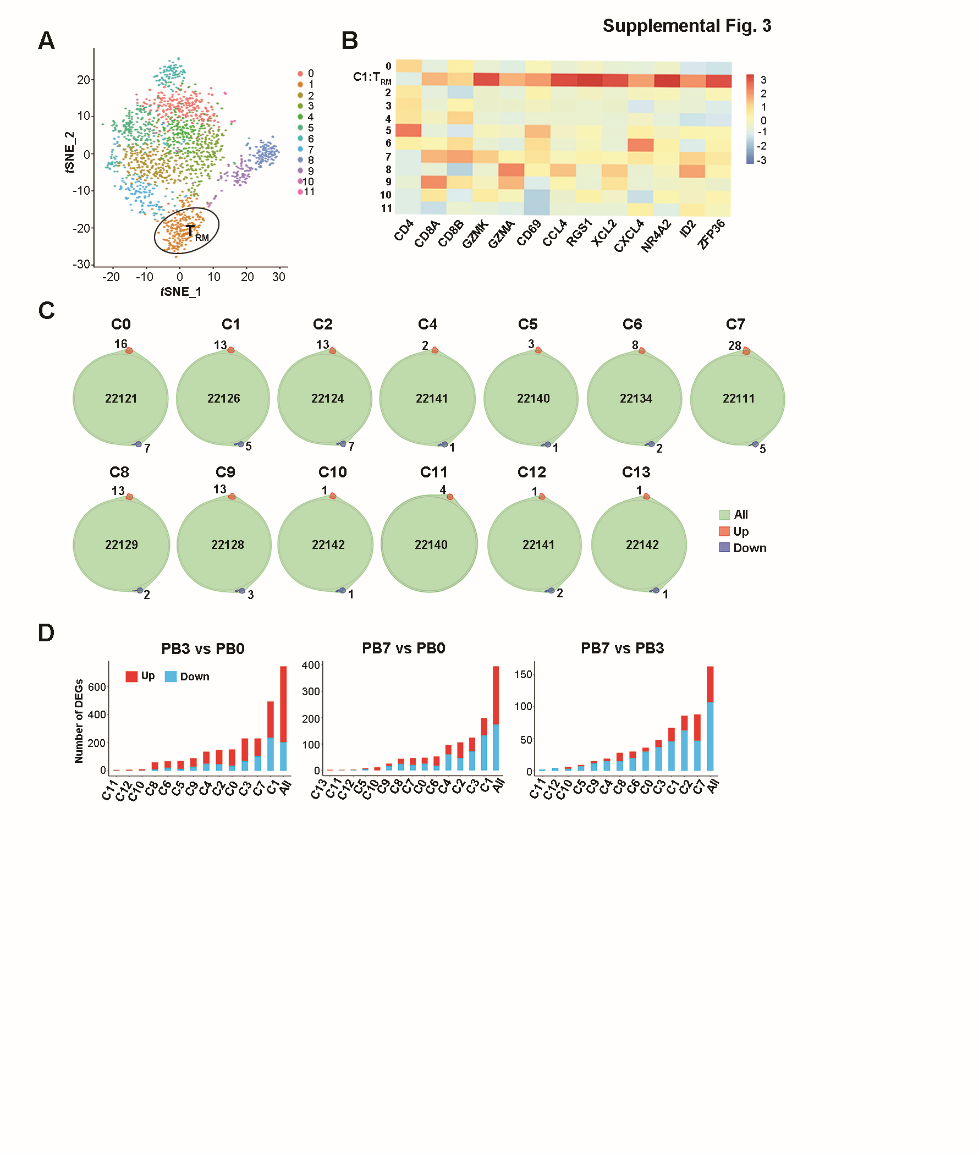


Supplemental Figure 4 T cell analysis excluding patient AL_D. A. Schematic of the sample collection in AL patients receiving Dara-BCD regimen (AL_D was excluded). BM aspirates and PB samples were taken at the same time. B. Pseudo-bulk analysis and clustering of individual PB and BM T cell samples (upper panel) and PB T cell samples alone (lower panel) collected before and after Dara-BCD. C. *t-*SNE visualization of PB and BM T cell clusters. D. Heatmap showing the row-scaled expression of selected signature genes for each cluster. E. Heatmap showing the row-scaled expression of *CD4*, *CD8A*, *CD8B*, *ZBTB16*, and *FOXP3*. F. Alterations of BM T cell subsets before (BM0) and after 3 or 7 cycles of Dara-BCD (BM3 and BM7). Each dot represents a cluster in a group of compare with the dot size representing -1*log10 (*P* value) and dot color representing cell ratio changes. G. Volcano plot showing DEGs in each cluster obtained from BM3 and BM0 comparison. The dot lines show 2-fold cutoff. H. Heatmap showing the row-scaled expression of signature genes for C1 (T_EMRA_) and C3 (T_RM_) cells.


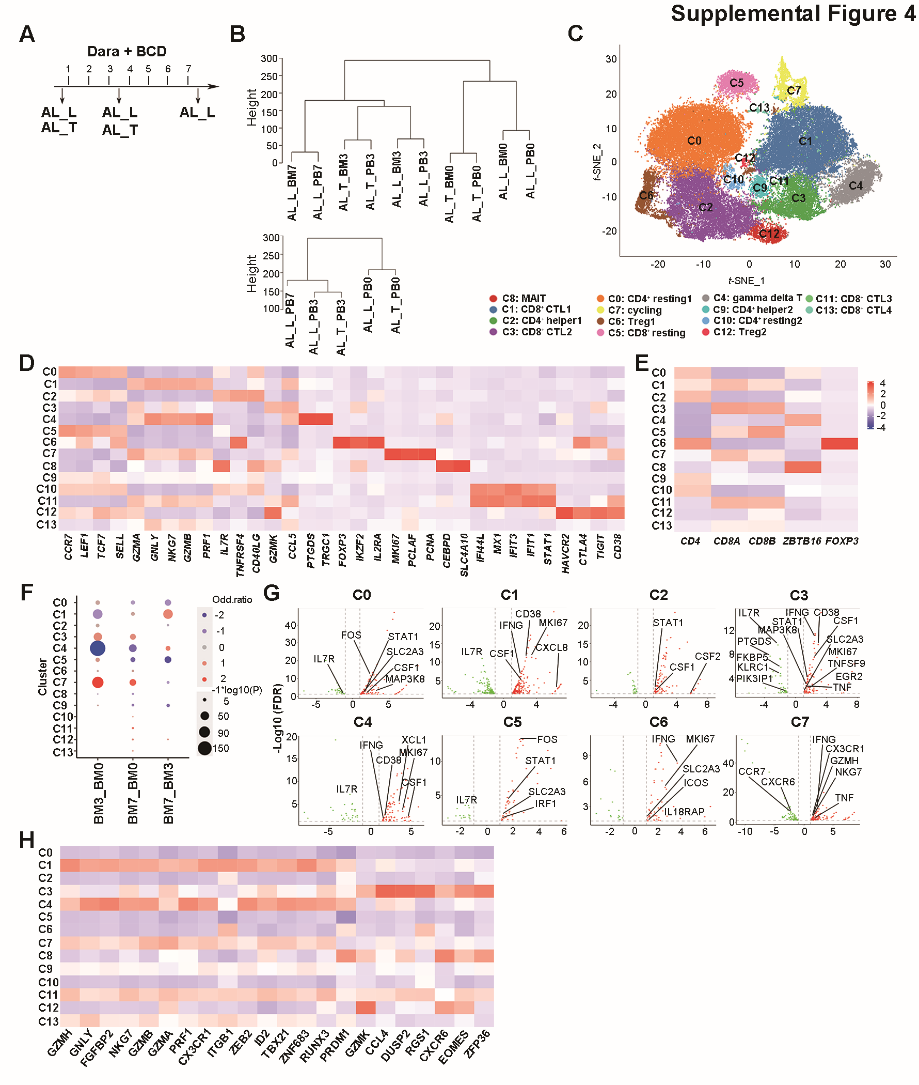

Supplement: Supplementary file 1 — SUPPORTING INFORMATION [file CTM2-11-e582-s002.docx]
